# Checklist of the family Ephydridae of Finland (Insecta, Diptera)

**DOI:** 10.3897/zookeys.441.7448

**Published:** 2014-09-19

**Authors:** Tadeusz Zatwarnicki, Jere Kahanpää

**Affiliations:** 1 Department of Biosystematics, Opole University, ul. Oleska 22, 45-052 Opole, Poland; 2 Finnish Museum of Natural History, Zoology Unit, P.O. Box 17, FI–00014 University of Helsinki, Finland

**Keywords:** Checklist, Finland, Diptera, Ephydridae

## Abstract

A checklist of 112 species of shore flies (Ephydridae, Diptera) recorded from Finland is presented. Comparing this to the list of Hackman (1980), 52 changes are made: 25 species are added (all but one recorded after 1980), 18 misidentifications are deleted, 5 junior synonyms are replaced and 5 updated generic combinations are given.

## Introduction

Shore flies (Ephydridae) are dark coloured, dull to shining, 1–6 mm long flies. Most species develop in aquatic or semi-aquatic conditions, feeding of algae, bacteria, yeasts or even decaying animal matter and excrements; or they are phytophagous and mining in leaves or stems. The group is divided into 5 subfamilies and 19 tribes (Zatwarnicki 1992), of which 15 could potentially be found in Finland.

Hackman (1980) originally listed 105 species. Zatwarnicki (1997) corrected the synonymy and generic placement of some species. Compared to Hackman (1980) the present list adds 25 species and removes 18 misidentified species (mostly *Hydrellia*). Five junior synonyms in *Hydrellia*, *Philygria*, *Scatella* and *Scatophila* are replaced; four species of *Ditrichophora* are combined with *Gymnoclasiopa* and one species of *Lamproscatella* with *Haloscatella*. Two species are endemic for Finland: *Psilopa nitidifacies* Frey, 1930 (note that this was omitted by Hackman 1980), and *Gymnoclasiopa psilopina* (Frey, 1933).

Nomenclature follows the current version of the Ephydridae section of Fauna Europaea (Zatwarnicki 2013). The subfamilies and the tribes are ordered systematically; genera within tribes and species within genera are listed alphabetically.

**Number of species:**

World: 1927 species and 63 nomina dubia (as of February 2014)

Europe: 340 species and 3 subspecies (Zatwarnicki 2013)

Finland: 112 species

Faunistic knowledge level in Finland: average

## Checklist

suborder Brachycera Macquart, 1834

clade Eremoneura Lameere, 1906

clade Cyclorrhapha Brauer, 1863

infraorder Schizophora Becher, 1882

clade Muscaria Enderlein, 1936

parvorder Acalyptratae Macquart, 1835

superfamily Ephydroidea Zetterstedt, 1837

**EPHYDRIDAE** Zetterstedt, 1837

DISCOMYZINAE Acloque, 1897

Discomyzini Acloque, 1897

***DISCOMYZA*** Meigen, 1830

*Discomyza incurva* (Fallén, 1823)

Psilopini Cresson, 1925

***PSILOPA*** Fallén, 1823

*Psilopa compta* (Meigen, 1830)

*Psilopa leucostoma* (Meigen, 1830)

*Psilopa marginella* Fallén, 1823

*Psilopa nigritella* Stenhammar, 1844

*Psilopa nitidifacies* Frey, 1958

*Psilopa nitidula* (Fallén, 1813)

*Psilopa polita* (Macquart, 1835)

***TRIMERINA*** Macquart, 1835

*Trimerina madizans* (Fallén, 1813)

*Trimerina microchaeta* Hendel, 1932

= *indistincta* Krivosheina 2004

HYDRELLIINAE Robineau-Desvoidy, 1830

Atissini Cresson, 1942

***ATISSA*** Haliday, 1839

*Atissa limosina* Becker, 1896

*Atissa pygmaea* Haliday, 1839

Hydrelliini Robineau-Desvoidy, 1830

***HYDRELLIA*** Robineau-Desvoidy, 1830

*Hydrellia albifrons* Fallén, 1813

*Hydrellia albilabris* (Meigen, 1830)

*Hydrellia cardamines* Haliday, 1839

= *baltica* Frey, 1930

*Hydrellia cochleariae* Haliday, 1839

= *flavicornis* misid.

*Hydrellia flaviceps* (Meigen, 1830)

= *discors* Collin, 1966

= *lapponica* misid.

*Hydrellia fulviceps* (Stenhammar, 1844)

= *chrysostoma* misid.

*Hydrellia fusca* (Stenhammar, 1844)

*Hydrellia griseola* (Fallén, 1813)

= *chrysostoma* (Meigen, 1830)

*Hydrellia laticeps* (Stenhammar, 1844)

*Hydrellia mutata* (Zetterstedt, 1846)

*Hydrellia obscura* (Meigen, 1830)

= *fascitibia* misid.

*Hydrellia pilitarsis* (Stenhammar, 1844)

*Hydrellia subalbiceps* Collin, 1966

*Hydrellia tarsata* Haliday, 1839

*Hydrellia thoracica* Haliday, 1839

= *modesta* misid.

Notiphilini Bigot, 1853

***DICHAETA*** Meigen, 1830

*Dichaeta caudata* (Fallén, 1813)

= *brevicauda* Loew, 1860

***NOTIPHILA*** Fallén, 1810

**sg. *Agrolimna*** Cresson, 1917

*Notiphila uliginosa* Haliday, 1839

**sg. *Notiphila*** Fallén, 1810

*Notiphila annulipes* Stenhammar, 1844

*Notiphila aquatica* Becker, 1896

*Notiphila brunipes* (Robineau-Desvoidy, 1830)

= *brunnipes* emend.

*Notiphila cinerea* Fallén, 1813

*Notiphila dorsata* Stenhammar, 1844

*Notiphila graecula* Becker, 1926

= *maculata* misid.

*Notiphila major* Stenhammar, 1844

*Notiphila pollinosa* Krivosheina, 1998

*Notiphila riparia* Meigen, 1830

GYMNOMYZINAE Latreille, 1829

Gymnomyzini Latreille, 1829

***ATHYROGLOSSA*** Loew, 1860

**sg. *Athyroglossa*** Loew, 1860

*Athyroglossa glabra* (Meigen, 1830)

***MOSILLUS*** Latreille, 1804

*Mosillus subsultans* (Fabricius, 1794)

Lipochaetini Becker, 1896

***GLENANTHE*** Haliday, 1839

*Glenanthe fuscinervis* Becker, 1896

= *ripicola* auct. nec Haliday, 1839

Hecamedini Mathis, 1991

***ALLOTRICHOMA*** Becker, 1896

*Allotrichoma bezzii* Becker, 1896

= *laterale* misid.

Ochtherini Dahl, 1959

***OCHTHERA*** Latreille, 1802

*Ochthera manicata* (Fabricius, 1794)

*Ochthera mantis* (De Geer, 1776)

Discocerinini Cresson, 1925

***DISCOCERINA*** Macquart, 1835

*Discocerina obscurella* (Fallén, 1813)

***DITRICHOPHORA*** Cresson, 1924

*Ditrichophora calceata* (Meigen, 1830)

*Ditrichophora fuscella* (Stenhammar, 1844)

***GYMNOCLASIOPA*** Hendel, 1930

*Gymnoclasiopa aurivillii* (Becker, 1896)

*Gymnoclasiopa bohemanni* (Becker, 1896)

*Gymnoclasiopa cinerella* (Stenhammar, 1844)

= *pulchella* misid.

*Gymnoclasiopa nigerrima* (Strobl, 1893)

*Gymnoclasiopa psilopina* (Frey, 1933)

***HECAMEDOIDES*** Hendel, 1917

*Hecamedoides glaucellus* (Stenhammar, 1844)

*Hecamedoides unispinosus* (Collin, 1943)

***POLYTRICHOPHORA*** Cresson, 1924

*Polytrichophora duplosetosa* (Becker, 1896)

ILYTHEINAE Cresson, 1943

Hyadinini Philips et al. in Cresson, 1949

***AXYSTA*** Haliday, 1839

*Axysta cesta* (Haliday, 1833)

***HYADINA*** Haliday, 1839

*Hyadina guttata* (Fallén, 1813)

*Hyadina humeralis* Becker, 1896

*Hyadina rufipes* (Meigen, 1830)

= *nitida* (Macquart, 1835)

*Hyadina scutellata* (Haliday, 1839)

***LYTOGASTER*** Becker, 1896

*Lytogaster abdominalis* (Stenhammar, 1844)

***NOSTIMA*** Coquillett, 1900

*Nostima picta* (Fallén, 1813)

***PELINA*** Haliday, 1839

*Pelina aenea* (Fallén, 1813)

*Pelina aenescens* (Stenhammar, 1844)

***PHILYGRIA*** Stenhammar, 1844

*Philygria femorata* (Stenhammar, 1844)

= *posticata* misid.

*Philygria flavipes* (Fallén, 1823)

*Philygria interstincta* (Fallén, 1813)

= *maculipennis* (Robineau-Desvoidy, 1830)

= *sexmaculata* Becker, 1896

*Philygria obtecta* Becker, 1896

*Philygria vittipennis* (Zetterstedt, 1838)

= *nigricauda* Stenhammar, 1844

= *trilineata* De Meijere, 1907

Ilytheini Cresson, 1943

***ILYTHEA*** Haliday, 1839

*Ilythea spilota* (Haliday in Curtis, 1832)

EPHYDRINAE Zetterstedt, 1837

Parydrini Wirth & Stone, 1956

***EUTAENIONOTUM*** Oldenberg, 1923

*Eutaenionotum guttipenne* (Stenhammar, 1843)

***PARYDRA*** Stenhammar, 1844

= ***Napaea*** Robineau-Desvoidy, 1830 preocc.

**sg. *Chaetoapnaea*** Hendel, 1930

*Parydra arctica* Clausen, 1971

*Parydra fossarum* (Haliday, 1833)

*Parydra mitis* (Cresson, 1930)

*Parydra nigritarsis* Strobl, 1893

*Parydra pusilla* (Meigen, 1830)

*Parydra quadripunctata* (Meigen, 1830)

**sg. *Parydra*** Stenhammar, 1844

*Parydra aquila* (Fallén, 1813)

*Parydra coarctata* (Fallén, 1813)

*Parydra nubecula* (Becker, 1896)

Ephydrini Zetterstedt, 1837

***CALOCOENIA*** Mathis, 1975

**sg. *Leptocoenia*** Mathis, 1975

*Calocoenia paurosoma* (Sturtevant & Wheeler, 1954)

***COENIA*** Robineau-Desvoidy, 1830

*Coenia curvicauda* (Meigen, 1830)

*Coenia palustris* (Fallén, 1823)

*Coenia vulgata* Krivosheina, 2001

***EPHYDRA*** Fallén, 1810

*Ephydra macellaria* Egger, 1862

*Ephydra riparia* Fallén, 1813

*Ephydra scholtzi* Becker, 1896

= *krogerusi* Frey, 1930

***PARACOENIA*** Cresson, 1935

*Paracoenia fumosa* (Stenhammar, 1844)

***SETACERA*** Cresson, 1930

*Setacera aurata* (Stenhammar, 1844)

*Setacera micans* (Haliday, 1833)

Scatellini Wirth & Stone, 1956

***HALOSCATELLA*** Mathis, 1979

*Haloscatella dichaeta* (Loew, 1860)

***LAMPROSCATELLA*** Hendel, 1917

*Lamproscatella sibilans* (Haliday, 1833)

***LIMNELLIA*** Malloch, 1925

*Limnellia fallax* (Czerny, 1903)

*Limnellia quadrata* (Fallén, 1813)

*Limnellia stenhammari* (Zetterstedt, 1846)

***PHILOTELMA*** Becker, 1896

*Philotelma defectum* (Haliday, 1833)

*Philotelma nigripenne* (Meigen, 1930)

***SCATELLA*** Robineau-Desvoidy, 1830

**sg. *Neoscatella*** Malloch, 1933

*Scatella crassicosta* Becker, 1896

*Scatella silacea* Loew, 1860

*Scatella subguttata* (Meigen, 1830)

**sg. *Scatella*** Robineau-Desvoidy, 1830

*Scatella obsoleta* Loew, 1861

= *callosicosta* Bezzi, 1895

*Scatella paludum* (Meigen, 1830)

*Scatella stagnalis* (Fallén, 1813)

*Scatella tenuicosta* Collin, 1930

***SCATOPHILA*** Becker, 1896

*Scatophila caviceps* (Stenhammar, 1844)

*Scatophila contaminata* (Stenhammar, 1844)

= *halterata* Becker, 1896

*Scatophila cribrata* (Stenhammar, 1844)

*Scatophila despecta* (Haliday, 1839)

= *hamifera* Becker, 1896

*Scatophila iowana* Wheeler, 1961

*Scatophila mesogramma* (Loew, 1869)

*Scatophila noctula* (Meigen, 1830)

= *flavitarsis* (Zetterstedt, 1846)

= *laevigata* (Loew, 1860)

= *silesiaca* Becker, 1896

*Scatophila quadriguttata* (Meigen, 1830)

= *variegata* (Loew, 1860)

## Excluded species

*Gymnoclasiopa aurifacies* (Strobl, 1893) misidentified

*Gymnoclasiopa plumosa* (Fallén, 1923) misidentified

*Hyadina nigricornis* Frey, 1930 not found within present borders

*Hydrellia argyrogenis* Becker, 1896 misidentified

*Hydrellia concolor* (Stenhammar, 1844) misidentified

*Hydrellia flavicornis* (Fallén, 1823) misidentified

*Hydrellia incana* (Stenhammar, 1844) misidentified

*Hydrellia meigeni* Zatwarnicki, 1988 misidentified

= *albiceps* (Meigen, 1830) preocc.

*Hydrellia nympheae* (Stenhammar, 1844) misidentified

*Hydrellia tibialis* Cresson, 1917 not found within present borders

= *diadema* Frey, 1930

*Notiphila stagnicola* (Robineau-Desvoidy, 1830) misidentified

*Psilopa pulicaria* (Haliday, 1839) misidentified

*Scatella lutosa* Haliday, 1833 misidentified

## Notes

***Gymnoclasiopa aurivillii* (Becker, 1896)** is probably a junior synonym of *Gymnoclasiopa nigerrima* (Strobl, 1893).

***Hydrellia***. At least two additional species occur in Finland but their proper names are still under investigation.
